# Designing optimal food intake patterns to achieve nutritional goals for Japanese adults through the use of linear programming optimization models

**DOI:** 10.1186/s12937-015-0047-7

**Published:** 2015-06-06

**Authors:** Hitomi Okubo, Satoshi Sasaki, Kentaro Murakami, Tetsuji Yokoyama, Naoko Hirota, Akiko Notsu, Mitsuru Fukui, Chigusa Date

**Affiliations:** 1 Department of Health Promotion, National Institute of Public Health, Saitama, Japan; 2 Department of Social and Preventive Epidemiology, School of Public Health, The University of Tokyo, Tokyo, Japan; 3 Department of Nutrition, School of Human Cultures, University of Shiga Prefecture, Shiga, Japan; 4 Graduate School of Health Science, Matsumoto University, Nagano, Japan; 5 Food Science and Nutrition Department, Tottori College, Tottori, Japan; 6 Department of Statistics, Osaka City University Medical School, Osaka, Japan; 7 Department of Food Science and Nutrition, School of Human Science and Environment, University of Hyogo, Hyogo, Japan

**Keywords:** Food intake pattern, Optimization, Linear programming, Japanese adults

## Abstract

**Background:**

Simultaneous dietary achievement of a full set of nutritional recommendations is difficult. Diet optimization model using linear programming is a useful mathematical means of translating nutrient-based recommendations into realistic nutritionally-optimal food combinations incorporating local and culture-specific foods. We used this approach to explore optimal food intake patterns that meet the nutrient recommendations of the Dietary Reference Intakes (DRIs) while incorporating typical Japanese food selections.

**Methods:**

As observed intake values, we used the food and nutrient intake data of 92 women aged 31–69 years and 82 men aged 32–69 years living in three regions of Japan. Dietary data were collected with semi-weighed dietary record on four non-consecutive days in each season of the year (16 days total). The linear programming models were constructed to minimize the differences between observed and optimized food intake patterns while also meeting the DRIs for a set of 28 nutrients, setting energy equal to estimated requirements, and not exceeding typical quantities of each food consumed by each age (30–49 or 50–69 years) and gender group.

**Results:**

We successfully developed mathematically optimized food intake patterns that met the DRIs for all 28 nutrients studied in each sex and age group. Achieving nutritional goals required minor modifications of existing diets in older groups, particularly women, while major modifications were required to increase intake of fruit and vegetables in younger groups of both sexes. Across all sex and age groups, optimized food intake patterns demanded greatly increased intake of whole grains and reduced-fat dairy products in place of intake of refined grains and full-fat dairy products. Salt intake goals were the most difficult to achieve, requiring marked reduction of salt-containing seasoning (65–80 %) in all sex and age groups.

**Conclusion:**

Using a linear programming model, we identified optimal food intake patterns providing practical food choices and meeting nutritional recommendations for Japanese populations. Dietary modifications from current eating habits required to fulfil nutritional goals differed by age: more marked increases in food volume were required in younger groups.

## Background

Adequate nutrient intake is important for the maintenance of health and prevention of chronic diseases [1]. Many countries have therefore set nutrient-based recommendations, generally referred to as Dietary Reference Intakes (DRIs), for the assessment and planning of dietary intake [2-4]. When considering the application of DRIs, it is necessary to design a food intake pattern that meets as many nutrient recommendations as possible while maintaining the intake of local and culture-specific foods. Food intake patterns meeting these requirements are useful in the development of practical and achievable dietary guidelines that promote healthy food choices.

Previous studies have suggested that the diet optimization method is useful in achieving these goals [5, 6]. Diet optimization by linear programming is a mathematical approach that optimizes (minimizes or maximizes) a linear function of decision variables while respecting multiple constraints. This methodology has been used to formulate nutritionally-optimal dietary patterns [7-12], to examine the relationship between diet cost and diet quality in Western countries [13-15], and to develop food-based dietary guidelines in developing countries [16]. A study in the Pacific Northwest of the USA generated sex-specific food plans that met both the key 2007 dietary recommendations for cancer prevention issued by the World Cancer Research Fund/American Institute of Cancer Research and the DRIs set by the Institute of Medicine [7]. Results showed that achieving cancer prevention goals required little modification of existing diets, but that fulfilling all nutrient recommendations required a large increase in food volume and a dramatic shift from existing diets. Such studies are necessary to identify the dietary modifications required to achieve all nutrient-based recommendations at the population level. To our knowledge, however, no comparable study has been reported in Asian countries, including Japan. The typical Japanese food intake pattern has characteristics seldom observed in Western and other populations, including high intake of refined grains, fish, seaweeds, soybean products, green tea, and salt and low intake of fat. Thus the nutritionally-optimal food intake patterns and the dietary modifications required may differ from those in Western and other populations [7, 12, 17]. Here, we applied a diet optimization model using linear programming to generate nutritionally-optimal food intake patterns that met the recommended DRIs [3] based on typical Japanese food selections.

## Methods

### Input dietary data for diet optimization

Input dietary data were obtained from an existing dataset derived from dietary records for 192 healthy Japanese adults aged 31–76 years who lived in three areas of Japan: Osaka (urban), Nagano (rural inland), and Tottori (rural coastal). These dietary records were taken on four non-consecutive days in each season of the year (16 days total) collected at intervals of approximately three months between November 2002 and September 2003. A detailed description of the study design and survey procedure has been published elsewhere [18, 19]. Written informed consent was obtained from each participant. A research protocol using the existing dietary dataset of this study was approved by the Ethics Committee at the Faculty of Medicine, The University of Tokyo (No. 3421). Dietary records were excluded for participants who had not completed the study (*n =* 8) or were older than 70 years of age (*n* = 10). Ultimately, dietary records obtained from 92 women aged 31–69 years and 82 men aged 32–69 years were used for the present analysis. Dietary data were stratified by sex and age group (<50 and ≥50 years) in accordance with the categorization of DRIs for Japanese adults [3], in which the standard value for each nutrient differed according to sex and age group.

Estimates of daily intake of food, energy, and selected nutrients were calculated based on the Standard Tables of Food Composition in Japan [20]. In the present study, a total of 1299 food items appeared in the dietary records. All food items were categorized into 19 food subgroups based on nutritional similarities and culinary usage. These food subgroups were further categorized into five major food groups (grains; vegetables; meat and alternatives; dairy products; and fruit) and a miscellaneous group in accordance with current dietary guidelines [21]. Classifications of food groups and subgroups are shown in Table 1.

**Table 1:** Food groups and subgroups (g/day) with lower or upper limit constraints included in the linear programming optimization models^a^

		Women				Men			
		30–49 years		50–69 years		30–49 years		50–69 years	
		(n = 45)		(n = 47)		(n = 40)		(n = 42)	
		Lower	Upper	Lower	Upper	Lower	Upper	Lower	Upper
Food group^b^	Subgroup^c^	P5	P95	P5	P95	P5	P95	P5	P95
Grains		250	562	278	522	368	929	421	724
	Whole grains^d^	-	125	-	125	-	164	-	164
	Refined grains	-	562	-	522	-	929	-	724
Vegetables		147	577	280	660	215	579	318	626
	Green and yellow vegetables	-	127	-	206	-	140	-	186
	Other vegetables	-	278	-	269	-	286	-	277
	Pulses (beans, soy, and nuts)	-	68	-	138	-	69	-	147
	Potatoes	-	112	-	103	-	123	-	115
	Mushrooms	-	21	-	33	-	28	-	30
	Seaweeds	-	22	-	45	-	21	-	32
Meat and alternatives		120	235	113	244	153	373	140	381
	Eggs	-	63	-	65	-	67	-	81
	Meat	-	102	-	71	-	165	-	118
	Fish	-	100	-	144	-	166	-	222
Dairy products		23	279	38	320	0.5	333	3	295
	Full-fat dairy products	-	260	-	320	-	319	-	278
	Reduced-fat dairy products^d^	-	203	-	203	-	215	-	215
Fruit		10	175	45	252	3	204	33	201
Others	Fats and oils	-	30	-	24	-	35	-	31
	Salt-containing seasoning	-	76	-	92	-	99	-	100
	Sugar and confectionary	-	93	-	115	-	115	-	89
	Alcoholic beverages	-	423	-	230	-	1039	-	1063
	Non-alcoholic beverages	-	1184	-	1374	-	1191	-	1375

^a^Diets assessed via four-day dietary records, one in each season (16 days in total), of residents in Osaka (urban), Nagano (rural inland) and Tottori (rural coast)

^b^In the five major food groups, constraints were set within the range from the 5th percentile (as a lower limit) to the 95th percentile (as an upper limit) of consumption by each sex and age group

^c^In food subgroups, however, constraints were set based on the 95th percentile of consumption by each sex and age group as an upper limit

^d^As intake of whole grain and reduced-fat dairy products were quite low among the study subjects, maximum intake level of each sex group was set as an upper limit for intakes of whole grain and reduced-fat dairy products, exceptionally

We established nutrient profiles for each food subgroup for energy plus 28 nutrients. To calculate these nutrient profiles, we assigned a weight to the nutrients from each representative food item that corresponded to the percentage consumption of its food subgroup, in accordance with previous studies [17, 22, 23]. Nutrient profiles were based on the nutrient content of 100 g of food from each food subgroup. Nutrient profiles were calculated separately for each sex and age group and were used as input data for our linear programming models.

### Objective function of linear programming models

Linear programming optimization is a mathematical tool for finding an optimal solution that minimizes or maximizes an objective function, which is dependent on a set of decision variables restricted by various linear constraints [5-7]. The objective function of the present study was to minimize the deviation in food intake between the observed and optimized food intake patterns such that the optimized food intake patterns met the nutritional recommendations with as little change as possible from the reported food intake. In accordance with previous studies [7, 24], the objective function was defined as the sum of the absolute value of the difference between the intake of each food subgroup in the optimized pattern and that in the observed food intake pattern divided by that in the observed food intake pattern (to standardize the difference across food subgroups), as follows:

$$ \mathrm{Y}={\displaystyle {\sum}_{i=1}^{i=19}\left|\left({\mathrm{X}}_{\mathrm{i}}^{\mathrm{opt}}\hbox{-} {\mathrm{X}}_{\mathrm{i}}^{\mathrm{obs}}\right)/\left.{\mathrm{X}}_{\mathrm{i}}^{\mathrm{obs}}\right|,\right.} $$

where Y denotes the objective function to minimize, X^opt^*i* denotes the quantity (g) of food subgroup *i* in the optimized food intake pattern, and X^obs^*i* denotes the mean quantity (g) of food subgroup *i* in the observed food intake pattern. The absolute value Y was nonlinear. Therefore, to apply linear programming, Y was transformed into a linear function using goal programming [25]. In accordance with previous studies [7, 23], new decision variables ≥0 and representing positive (*P*1 to *P*19) and negative (*N*1 to *N*19) deviation from the observed food subgroup quantity were created and defined as follows:

if X^opt^_*i*_ < X^obs^_*i*_, then *N*_*i*_ = (X^obs^_*i*_ - X^opt^_*i*_) / X^obs^_*i*_ and *P*_*i*_ = 0.

if X^opt^_*i*_ > X^obs^_*i*_, then *N*_*i*_ = 0 and *P*_*i*_ = (X^opt^_*i*_ - X^obs^_*i*_) / X^obs^_*i*_.

if X^opt^_*i*_ = X^obs^_*i*_, then *N*_*i*_ = 0 and *P*_*i*_ = 0.

Subject to: *P*_*i*_ - *N*_*i*_ = (X^opt^_*i*_ - X^obs^_*i*_) / X^obs^_*i*_.

The new linear function called Y' was expressed as the sum of the deviational variables and minimized as follows:

$$ \mathrm{Y}\hbox{'}={\displaystyle {\sum}_{i=1}^{i=19}{P}_{\mathrm{i}}}+{N}_{\mathrm{i}}. $$

Each food subgroup in the objective function was linked to the nutrient profile database established for this study. This model calculated the intake of food groups and subgroups at all times and checked whether nutritional constraints (as mentioned below) were fulfilled. The observed intake of each food group or subgroup was defined according to the mean intake of that food group or subgroup across the whole population.

### Nutritional constraints for linear programming models

Food intake patterns optimized by linear programming models should not only minimize the gap between observed and optimized food intake patterns but should also be practical and provide desirable energy and nutrient intake levels. We therefore introduced a set of constraints to the linear programming models (Tables 1 and 2). The solution selected by a model must satisfy all of the specified constraints.

**Table 2:** List of nutritional constraints included in the linear programming optimization models

	Women^a^				Men^a^			
Dietary Reference Intake constraints	30–49 years		50–59 years		30–49 years		50–59 years	
Estimated Energy Requirement (kcal/day)^b^	2000		1900		2650		2450	
Nutrients with DG								
Total fat (% of energy)	20–30		20–30		20–30		20–30	
SFA (% of energy)	≤7		≤7		≤7		≤7	
Carbohydrate (% of energy)	50–65		50–65		50–65		50–65	
Dietary fibre (g/day)	≥18		≥18		≥20		≥20	
Sodium (salt-equivalent) (g/day)^c^	<7.0		<7.0		<8.0		<8.0	
Potassium (mg/day)	≥2600		≥2600		≥3000		≥3000	
Nutrients with RDA or AI	RDA or AI	UL	RDA or AI	UL	RDA or AI	UL	RDA or AI	UL
Protein (g/day)	≥50	-	≥50	-	≥60	-	≥60	-
n-3 PUFA (g/day)	≥1.6	-	≥2.0	-	≥2.1	-	≥2.4	-
n-6 PUFA (g/day)	≥8	-	≥8	-	≥10	-	≥10	-
Vitamin A (μgRAE/day)^d^	≥700	≤2700	≥700	≤2700	≥900	≤2700	≥850	≤2700
Vitamin D (μg/day)	≥5.5	≤100	≥5.5	≤100	≥5.5	≤100	≥5.5	≤100
Vitamin E (mg/day)^e^	≥6.0	≤700	≥6.0	≤700	≥6.5	≤900	≥6.5	≤850
Vitamin K (μg/day)	≥150	-	≥150	-	≥150	-	≥150	-
Vitamin B_1_ (mg/day)	≥1.1	-	≥1.0	-	≥1.4	-	≥1.3	-
Vitamin B_2_ (mg/day)	≥1.2	-	≥1.1	-	≥1.6	-	≥1.5	-
Niacin (mgNE/day)^f^	≥12	-	≥11	-	≥15	-	≥14	-
Vitamin B_6_ (mg/day)	≥1.2	-	≥1.2	-	≥1.4	-	≥1.4	-
Vitamin B_12_ (μg/day)	≥2.4	-	≥2.4	-	≥2.4	-	≥2.4	-
Folate (μg/day)	≥240	-	≥240	-	≥240	-	≥240	-
Pantothenic acid (mg/day)	≥4	-	≥5	-	≥5	-	≥5	-
Vitamin C (mg/day)	≥100	-	≥100	-	≥100	-	≥100	-
Calcium (mg/day)	≥650	≤2500	≥650	≤2500	≥650	≤2500	≥700	≤2500
Magnesium (mg/day)	≥290	-	≥290	-	≥370	-	≥350	-
Phosphorus (mg/day)	≥800	≤3000	≥800	≤3000	≥1000	≤3000	≥1000	≤3000
Iron (mg/day)^g^	≥10.5	≤40	≥6.5	≤40	≥7.5	≤55	≥7.5	≤50
Zinc (mg/day)	≥8	≤35	≥8	≤35	≥10	≤45	≥10	≤45
Copper (mg/day)	≥0.8	≤10	≥0.8	≤10	≥1.0	≤10	≥0.9	≤10
Manganese (mg/day)	≥3.5	≤11	≥3.5	≤11	≥4.0	≤11	≥4.0	≤11

*DG*, Tentative dietary goal for preventing life-style related disease; *SFA*, Saturated fatty acids; *RDA*, Recommended dietary allowance; *AI*, Adequate intake; *UL*, Tolerable upper intake level; *PUFA*, Polyunsaturated fatty acids; RAE, Retinol activity equivalent

^a^Sex and age categorizations were according to the Dietary Reference Intakes for Japanese, 2015

^b^Values of Estimated Energy Requirement are based on physical activity level II (moderate)

^c^For convenience, the DG for sodium are expressed as referring to salt-equivalent [salt (g) = 58.5/23 × sodium (g)]

^d^1 μgRAE = retinol (μg) + β-carotene (μg) × 1/12 + α-carotene (μg) × 1/24 + β-cryptoxantin (μg) × 1/24 + other provitamin A carotenoides (μg) × 1/24

^e^Computation was made on α-tocopherol. Vitamin E other than α-tocopherol was not included

^f^Niacin equivalents were computed as niacin (mg) + tryptophan (mg)/60

^g^Value was the RDA of iron for menstruating women aged 30–49 years

Food use constraints set upper limits on the quantity of each food to ensure that program models did not exceed the amounts usually consumed by a particular population. These limits were derived from the actual intake patterns for each sex and age group as reported through 16-day dietary records (Table 1). Specifically, the dietary intake of all foods from each food subgroup was required not to exceed the 95th percentile of intake for each sex and age group. In the present population of Japanese adults, however, intake of whole grains and reduced-fat dairy products was very low. For these subgroups, therefore, we exceptionally set a maximum intake level for each sex. For the five food groups, as for the subgroups, we limited dietary intake to within the range from the 5th percentile to the 95th percentile of observed intake for each sex and age group.

Nutritional constraints were included to ensure that the nutritional content of each optimized food intake pattern was equal to or greater than the desired value, which was based on the DRIs for Japanese adults [3]. Energy constraints were set to ensure that the energy content of the optimized food intake pattern was equal to the Estimated Energy Requirement (EER) for each sex and age group. According to their self-reported physical activity levels (low, moderate, and high), which were assessed by questionnaire [18], most subjects were classified as having a moderate physical activity level. In accordance with the EERs published with the Japanese DRIs for persons engaging in moderate physical activity [3], EER values were set at 2000 kcal/day for women aged 30–49 years, 1900 kcal/day for women aged 50–69 years, 2650 kcal/day for men aged 30–49 years, and 2450 kcal/day for men aged 50–69 years [3]. For nutrients with Recommended Dietary Allowance (RDA) or Adequate Intake (AI), namely protein, *n*-3 polyunsaturated fatty acids (PUFA), *n*-6 PUFA, vitamin A expressed as retinol activity equivalent (RAE), vitamin D, vitamin E expressed as α-tocopherol, vitamin K, vitamin B_1_, vitamin B_2_, niacin (expressed as niacin equivalent), vitamin B_6_, vitamin B_12_, folate, pantothenic acid, vitamin C, calcium, magnesium, phosphorus, iron, zinc, copper, and manganese, RDA or AI was the criterion by which we determined whether each nutritional goal had been met by the optimized food intake pattern. For nutrients with Tentative Dietary Goal for Preventing Life-style-related Disease (DG) such as total fat, saturated fatty acids (SFA), carbohydrate, dietary fibre, sodium (expressed as salt-equivalent), and potassium, the DG values were used as a nutritional constraint. Tolerable Upper Intake Level (UL), if defined, was also used to create acceptable nutrient ranges.

### Statistical analysis

We used SAS statistical software version 9.4 (SAS Institute Inc., Cary, NC, USA) to merge and arrange our data, which were then transferred to Excel files. Microsoft EXCEL SOLVER (Frontline Systems) was used for linear programming in accordance with the method described by Briend *et al.* [5].

## Results

The characteristics of the subjects who provided our dietary data have been previously reported [18, 19]. Mean age, energy intake, and the ratio of reported energy intake to EER (EI/EER) of input dietary data (i.e. observed dietary intake data) for linear programming models were as follows: 39.3 years, 1856 kcal/day, and 0.93, respectively, for women aged 30–49 years (*n* = 45); 59.4 y, 1898 kcal/day, and 0.97 for women aged 50–69 years (*n* = 47); 40.9 y, 2391 kcal/day, and 0.90 for men aged 30–49 years (*n* = 40); and 59.6 y, 2457 kcal/day, and 1.00 for men aged 50–69 years (*n* = 42).

Mathematically optimized food intake patterns satisfying all nutritional constraints were obtained for each sex and age group using the linear programming model. Table 3 shows a comparison of nutrient contents between the observed and optimized food intake patterns. The number of nutrients for which nutritional goals were not achieved in the observed food intake pattern was 13 for women aged 30–49 years, four for women aged 50–69 years, 13 for men aged 30–69 years, and six for men aged 50–69 years. In contrast, all optimized food intake patterns achieved the nutritional goals of the DRIs for all nutrients. It should be noted, however, that all optimized diets generated in the present analysis contained exactly 100 % of the salt upper limit proposed by the DRIs, suggesting that salt-equivalent is a limiting nutrient for all sex and age groups. The other limiting nutrients in the present models were SFA and iron for women aged 30–49 years; SFA, total dietary fibre, and vitamin B_1_ for women aged 50–69 years; n-6 PUFA, total dietary fibre, retinol activity equivalent, vitamin B_1,_ and magnesium for men aged 30–69 years; and total dietary fibre and retinol activity equivalent for men aged 50–69 years.

**Table 3:** Comparison of nutrient contents between the observed and optimized daily food intake patterns

		Women				Men			
		30–49 years (n = 45)		50–69 years (n = 47)		30–49 years (n = 40)		50–69 years (n = 42)	
	Unit/day	Observed^b^	Optimized	Observed^b^	Optimized	Observed^b^	Optimized	Observed^b^	Optimized
Energy	kcal	1856	2000	1898	1900	2391	2650	2457	2450
Protein	g	66.8	82.6	74.9	73.1	81.3	104.9	89.5	89.3
Total fat	%E	29.1	23.0	26.0	25.5	26.2	23.5	24.0	23.5
SFA	%E	8.5^a^	7.0	7.2^a^	7.0	7.3^a^	6.1	6.3	6.1
n-3 PUFA	g	2.3	1.9	2.7	2.7	2.8	3.2	3.3	3.2
n-6 PUFA	g	10.7	8.1	10.2	10.1	12.7	10.0	11.9	12.0
Carbohydrate	%E	53.8	59.1	56.7	57.8	53.1	55.7	54.4	54.9
Total dietary fibre	g	13.1^a^	21.2	17.3	18.0	13.6^a^	20.0	17.5^a^	20.0
Vitamin A	μgRAE	584^a^	804	682^a^	700	628^a^	900	786^a^	850
Vitamin D	μg	6.2	8.1	10.2	10.2	7.4	13.1	12.0	11.9
Vitamin E^d^	mg	7.1	7.4	8.1	8.3	8.0	9.3	9.0	9.9
Vitamin K	μg	211	308	279	292	212	292	283	311
Vitamin B_1_	mg	0.87^a^	1.3	0.96^a^	1.00	1.1^a^	1.4	1.1^a^	1.3
Vitamin B_2_	mg	1.2	1.7	1.5	1.4	1.4^a^	1.7	1.6	1.7
Niacin^e^	mgNE	15.9	36.4	18.0	30.0	20.9	45.8	23.1	40.3
Vitamin B_6_	mg	1.1^a^	1.7	1.4	1.4	1.4^a^	1.9	1.7	1.9
Vitamin B_12_	μg	6.5	8.3	9.1	8.8	8.0	13.1	11.5	11.4
Folate	μg	309	469	423	431	339	475	455	473
Pantothenic acid	mg	5.8	7.9	6.5	6.6	6.8	8.6	7.5	8.2
Vitamin C	mg	90^a^	150	141	148	92^a^	158	138	147
Salt equivalent^f^	g	12.2^a^	7.0	12.4^a^	7.0	16.2^a^	8.0	14.5^a^	8.0
Potassium	mg	2393^a^	3403	3049	3004	2672^a^	3595	3209	3346
Calcium	mg	526^a^	674	656	650	544^a^	677	641^a^	700
Magnesium	mg	249^a^	385	310	310	286^a^	370	348^a^	401
Phosphorus	mg	1015	1337	1173	1166	1197	1520	1350	1492
Iron	mg	7.4^a^	10.5	9.5	9.1	8.3	10.1	10.3	10.7
Zinc	mg	7.9^a^	10.1	8.6	8.7	9.6^a^	12.1	10.4	10.9
Copper	mg	1.1	1.5	1.3	1.3	1.3	1.7	1.5	1.6
Manganese	mg	3.1^a^	5.0	4.3	4.6	3.8^a^	4.9	4.8	5.7
Alcohol	%E	1.5	1.4	0.9	0.9	5.4	5.0	5.8	6.2

^a^Nutrients not meeting the DRIs

^b^Observed intake of nutrients in each sex and age group was based on the average population intake of nutrients

^c^1 μgRAE = retinol (μg) + β-carotene (μg) × 1/12 + α-carotene (μg) × 1/24 + β-cryptoxantin (μg) × 1/24 + other provitamin A carotenoides (μg) × 1/24

^d^Computation was made on α-tocopherol. Vitamin E other than α-tocopherol was not included

^e^Niacin equivalents were computed as niacin (mg) + tryptophan (mg)/60

^f^For convenience, the DG of sodium are expressed as referring to salt-equivalent [salt (g) = 58.5/23 × sodium (g)]

Table 4 shows a comparison of food quantity between the observed and optimized food intake patterns. For convenience in data interpretation, we assumed that dietary modification (either increase or decrease) was required when the difference between the observed and optimized food intake patterns was more than 10 or −10 %. In the younger age groups (30–49 years), large modifications were required to increase the intake of food items in the following food groups: vegetables, which needed to be increased by 65 % for women and 55 % for men, particularly green and yellow vegetables, other vegetables, pulses, and seaweeds for both sexes and mushrooms for women; meat and alternatives, which needed to be increased by 38 % for women and 47 % for men, particularly eggs for women and fish for men; and fruit, which needed to be increased by 96 % for women and 172 % for men. Furthermore, the intake required to satisfy all nutritional constraints reached the upper limit (95th percentile of observed intake) in several food groups, reflecting the fact that their consumption by this sample was low; these were vegetables, including green and yellow vegetables and other vegetables, for both sexes; pulses, seaweeds, and meat and alternatives including eggs for women; and fish for men. In contrast, little modification of the existing diet was required in the older age groups (50–69 years), particularly in women, although increased intake of green and yellow vegetables for men (25 %) and increased intake of other vegetables for women (14 %) were still required. Across all sex and age groups, optimized food intake patterns called for greatly increased intake of whole grains and reduced-fat dairy products in place of intake of refined grains and full-fat dairy products. The optimized food intake pattern also called for a marked reduction (by 65–80 %) in salt-containing seasoning for all sex and age groups in order to keep salt consumption within the limits.

**Table 4:** Comparison of food amounts (g/day) between the observed and the optimized food intake patterns

	Women				Men			
	30–49 years (n = 45)		50–69 years (n = 47)		30–49 years (n = 40)		50–69 years (n = 42)	
	Observed^a^	Optimized	Observed^a^	Optimized	Observed^a^	Optimized	Observed^a^	Optimized
Grains	401	393	396	426	564	642	567	580
Whole grains	4	125^b^	12	57	2	35	14	164^b^
Refined grains	397	267	384	369	562	607	554	416
Vegetables	349	577^b^	486	522	372	579^b^	489	526
Green and yellow vegetables	77	127^b^	125	134	79	140^b^	118	148
Other vegetables	147	278^b^	188	214	155	286^b^	199	206
Pulses	44	68^b^	76	76	44	54	71	71
Potatoes	61	66	64	64	73	73	70	70
Mushroom	9	16	14	14	10	10	14	14
Seaweeds	11	22^b^	18	18	11	16	16	16
Meat and alternatives	170	235^b^	182	182	225	329	241	241
Eggs	38	63^b^	37	37	45	45	46	46
Meat	69	88	48	48	97	118	71	71
Fish	63	83	97	97	83	166^b^	124	124
Milk products	138	134	165	164	110	110	124	174
Full-fat dairy products	121	0	130	120	100	73	101	71
Reduced-fat dairy products	17	134	35	44	10	36	24	103
Fruit	89	175^b^	144	144	75	204^b^	115	115
Others								
Fats and oils	19	0	15	15	24	9	19	19
Salt-containing seasoning	55	18	63	22	66	13	71	23
Sugars and confectionary	59	93	65	65	48	48	50	50
Alcoholic beverages	83	83	48	48	322	322	311	311
Non-alcoholic beverages	750	1184	808	808	773	885	796	796

^a^Observed intakes of food groups and subgroups by each sex and age group based on average population intake of food group and subgroup

^b^95th percentile upper constraint reached for the food group and food subgroup

## Discussion

Using the linear programming model, we mathematically obtained sex- and age-specific optimized food intake patterns that achieved a set of 28 nutrient recommendations given in the DRIs for Japanese adults. The present study demonstrates how nutrient-based recommendations can be translated into nutritionally adequate food intake patterns with minimal modification of current dietary habits among Japanese adults. To our knowledge, the application of mathematical diet optimization models by linear programming to develop recommended food intake patterns in an Asian population has not been described before.

With the exception of fruit and vegetable intake among the younger age groups, our optimized food intake patterns did not strongly differ from the observed intake patterns at the food group level (Table 4). At the food subgroup level, in contrast, achieving nutritional goals required substantial dietary modifications, namely, an increase in whole grains of more than 10-fold, an increase in reduced-fat dairy products of 26–705 %, a decrease in full-fat dairy products of 7.8–100 %, and a decrease in salt-containing seasoning of 65–80 %. These results were generally consistent with food choices recommended by the dietary guidelines developed in Western countries [9, 26-28]. However, the current Japanese dietary guidelines make no mention of such dietary modifications (i.e. increased whole grains and reduced-fat dairy products and decreased full-fat dairy products and salt-containing seasoning), probably due to insufficient evidence [21]. The present findings might therefore facilitate the revision of these guidelines.

Although the nutritional constraints set by the DRIs did not differ substantially between younger and older age groups (except in the case of iron among women), the dietary modifications necessary to achieve the established nutritional goals differed according to age group: more marked increases in food volume were required in younger age groups. In older age groups, on the other hand, observed intake of most food groups and subgroups was already very close to optimized intake, except for dairy products in men. Furthermore, the number of nutrients for which nutritional goals were already satisfied by the observed food intake patterns was higher in older age groups (24 for women and 22 for men) than in younger age groups (15 for both sexes). These results are partially consistent with those of a previous study in a large US population showing that sodium levels were closer to the proposed guideline in the observed diets of adults aged >50 years, particularly men, than in those of younger adults aged 20–30 or 30–50 years [12]. The reasons for these differences in the degree of dietary modification required for each age group are unclear, but they might be at least partially explained by age-specific dietary habits and differences in dietary awareness. Indeed, according to the National Health and Nutrition Survey in Japan, older people tended to pay more attention to their diets than younger people did [29], and the percentages of subjects who evaluated their current diets as “excellent/good” were 57.4, 60.4, 63.5, and 79.6 % among women aged 30–39 years, 40–49 years, 50–59 years, and 60–69 years, respectively, and 67.1, 68.3, 76.2, and 83.9 % among men aged 30–39 years, 40–49 years, 50–59 years, and 60–69 years, respectively [30]. Few studies have examined the differences in necessary diet modifications according to age, a fact which hinders comparison of our results with those of other studies. Further study is therefore required to confirm whether this tendency is consistently observed in other populations.

Here, we demonstrated that, for younger age groups, meeting nutritional goals requires a drastic increase in consumption of specific food groups and food subgroups such as green and yellow vegetables, other vegetables, and fruit for both sexes; pulses, seaweeds, and eggs for women; and fish for men. Consequently, in our optimized diets, these food groups and subgroups reached the upper boundary limits for this study population, while fats and oils reached the lower limits among young women. However, whether such substantial increases in food consumption are even feasible at the population level remains questionable, as the achievement of goals for specific nutrients, such as iron and sodium, within the stringent consumption constraints required may be difficult. The present findings in younger age groups should therefore be interpreted carefully, and the issue of feasibility must be addressed.

Previous studies using diet optimization models have found that achieving nutritional goals with regard to population- or culture-specific nutrients is difficult [7, 9, 11, 17, 24, 26]. For the American population, for example, intake of vitamin E at all energy levels, potassium at lower energy levels, and sodium at higher energy levels did not meet nutritional goals [7, 12, 17, 26]. For the French population, the key problem nutrients were vitamin D, magnesium, sodium, and SFA for both sexes, cholesterol for men, and iron, calcium, and vitamin E for women [9, 24]. In the present study, as in similar studies from other countries, salt equivalent (sodium) was the most difficult constraint to fulfil for all sex and age groups. In addition, iron for young women only, SFA for all women, and total dietary fibre and vitamin A for men were also identified as limiting nutrients. The fact that different limiting nutrients have been identified in different countries might be explained by differences not only in dietary habits but also in dietary standards. For example, the recommended intake of α-tocopherol in Japan is 6.0–6.5 mg/day [3], compared to 15 mg/day in the USA and Canada [2] and 12 mg/day in France [9]. Recommendations for dietary fibre, potassium, calcium, and magnesium are also substantially lower in Japan than in the USA and France [2, 9]. As part of the present study, we attempted to formulate optimal food intake patterns for Japanese adults, using typical Japanese food selections, that would achieve the DRIs used in the USA/Canada as an experiment on the application of linear programming models. However, we were unable to generate food intake patterns that met these criteria. This outcome shows that the optimal solution for a given population might be dependent on the reference values selected as nutritional constraints. Therefore, suitable and achievable country-specific dietary goals should be selected with consideration for the realistic consumption levels of each population when linear dietary optimized models are used to generate nutritionally-optimal food intake patterns.

Several limitations to the present study warrant mention. First, the study subjects may not have been representative because they were not randomly sampled from the general Japanese population; rather, they were volunteers. Moreover, the participants might be highly health-conscious because almost all of them completed the study despite the strict study design. Second, the sample size was relatively small for both women (*n =* 92) and men (*n =* 82). Therefore, our estimates of food and nutrient intake, particularly the distribution of food consumption levels, might not be stable. Third, the present analysis was based on the secondary dietary data which was collected during 2002–2003. The stability of food intake pattern and change in dietary habits during the time gap between data collection and analysis might have slightly influenced the conclusion. Additional studies using latest dietary data are therefore needed to confirm our findings. Fourth, reporting errors such as under- or over-reporting could not be avoided because, as in many other studies [31], dietary intake information was self-reported. This may induce bias when reported dietary intake levels are compared with corresponding optimized dietary intake (because the latter does not consider this problem). Nevertheless, EI/EER values in the present study were close to 1.00 (0.90–1.00), suggesting sufficient accuracy at least at the group level. Additionally, the results were not materially changed when food and nutrient intakes were adjusted for EI and then standardized to EER in consideration of possible under- or over-reporting of intake. Fifth, the validity of results obtained using diet optimization models is dependent on the accuracy of the model’s simulation and the quality of the input data. In addition, the linear programming optimization model generated only a single food intake pattern for each sex and age group, and variation due to individual food choices was not taken into account. Finally, and most importantly, the reliability of the DRI for each nutrient, which is used as a nutrient constraint in linear programming models, is dependent on the accuracy of the available studies on that nutrient [3].

In conclusion, diet optimization using linear programming models can effectively translate nutrient-based recommendations into realistic food intake patterns for a Japanese population. Substantial dietary modification was required to increase the intake of whole grains and reduced-fat dairy products as well as fruit and vegetables, and to decrease that of full-fat dairy products and salt-containing seasoning to meet nutrient recommendations. Further studies are required to confirm our observations in a more representative sample of the Japanese population and to examine the application of linear programming optimization models in other Asian populations.

## Abbreviations

DRIs: Dietary reference intakes
EER: Estimated energy requirement
PUFA: Polyunsaturated fatty acids
RDA: Recommended dietary allowance
AI: Adequate intake
RAE: Retinol activity equivalent
DG: Tentative dietary goal for preventing life-style-related disease
SFA: Saturated fatty acids
UL: Tolerable upper intake level

## References

[1] World Health Organization (2003). Diet, Nutrition and the Prevention of Chronic Diseases. Joint WHO/FAO Expert Consultation. WHO technical report series no. 916.

[2] Institute of Medicine, Food and Nutrition Board (2000). Dietary Reference Intakes: Applications in Dietary Assessment.

[3] Ministry of Health, Labour, and Welfare of Japan. Dietary Reference Intakes for Japanese, 2015. [in Japanese]. http://www.mhlw.go.jp/file/05-Shingikai-10901000-Kenkoukyoku-Soumuka/0000083869.pdf. (Accessed May 10, 2015).

[4] Doets EL, de Wit LS, Dhonukshe-Rutten RA, Cavelaars AE, Raats MM, Timotijevic L (2008). Current micronutrient recommendations in Europe: towards understanding their differences and similarities. Eur J Nutr.

[5] Briend A, Darmon N, Ferguson E, Erhardt JG (2003). Linear programming: a mathematical tool for analyzing and optimizing children’s diets during the complementary feeding period. J Pediatr Gastroenterol Nutr.

[6] Soden PM, Fletcher LR (1992). Modifying diets to satisfy nutritional requirements using linear programming. Br J Nutr.

[7] Masset G, Monsivais P, Maillot M, Darmon N, Drewnowski A (2009). Diet optimization methods can help translate dietary guidelines into a cancer prevention food plan. J Nutr.

[8] Ferguson EL, Darmon N, Fahmida U, Fitriyanti S, Harper TB, Premachandra IM (2006). Design of optimal food-based complementary feeding recommendations and identification of key “problem nutrients” using goal programming. J Nutr.

[9] Maillot M, Vieux F, Amiot MJ, Darmon N (2010). Individual diet modeling translates nutrient recommendations into realistic and individual-specific food choices. Am J Clin Nutr.

[10] Kersting M, Alexy U, Clausen K (2005). Using the concept of Food Based Dietary Guidelines to Develop an Optimized Mixed Diet (OMD) for German children and adolescents. J Pediatr Gastroenterol Nutr.

[11] Gao X, Wilde PE, Lichtenstein AH, Bermudez OI, Tucker KL (2006). The maximal amount of dietary alpha-tocopherol intake in U.S. adults (NHANES 2001–2002). J Nutr.

[12] Maillot M, Drewnowski A (2012). A conflict between nutritionally adequate diets and meeting the 2010 dietary guidelines for sodium. Am J Prev Med.

[13] Darmon N, Ferguson EL, Briend A (2006). Impact of a cost constraint on nutritionally adequate food choices for French women: an analysis by linear programming. J Nutr Educ Behav.

[14] Darmon N, Ferguson EL, Briend A (2002). A cost constraint alone has adverse effects on food selection and nutrient density: an analysis of human diets by linear programming. J Nutr.

[15] Carlson A, Lino M, Juan W, Hanson K, Basiotis P (2007). Thrifty Food Plan, 2006. (CNPP-19).

[16] Ferguson EL, Darmon N, Briend A, Premachandra IM (2004). Food-based dietary guidelines can be developed and tested using linear programming analysis. J Nutr.

[17] Gao X, Wilde PE, Lichtenstein AH, The TKL (2005). USDA Food Guide Pyramid is associated with more adequate nutrient intakes within energy constraints than the 1992 Pyramid. J Nutr.

[18] Okubo H, Sasaki S, Hirota N, Notsu A, Todoriki H, Miura A (2006). The influence of age and body mass index on relative accuracy of energy intake among Japanese adults. Public Health Nutr.

[19] Okubo H, Murakami K, Sasaki S, Kim MK, Hirota N, Notsu A (2010). Relative validity of dietary patterns derived from a self-administered diet history questionnaire using factor analysis among Japanese adults. Public Health Nutr.

[20] Science and Technology Agency (2010). Standard Tables of Food Composition in Japan 2010.

[21] Yoshiike N, Hayashi F, Takemi Y, Mizoguchi K, Seino F. A new food guide in Japan: the Japanese food guide Spinning Top. Nutr Rev. 2007;65:149–54. 10.1111/j.1753-4887.2007.tb00294.x 17503709

[22] Marcoe K, Juan W, Yamini S, Carlson A, Britten P (2006). Development of food group composites and nutrient profiles for the MyPyramid Food Guidance System. J Nutr Educ Behav.

[23] Alexy U, Kersting M, Sichert-Hellert W (2001). The “Optimized Mixed Diet”: Evaluation of a Food Guide System for Children and Adolescents. J Nutr Educ.

[24] Maillot M, Vieux F, Ferguson EF, Volatier JL, Amiot MJ, Darmon N (2009). To meet nutrient recommendations, most French adults need to expand their habitual food repertoire. J Nutr.

[25] Anderson AM, Earle MD (1983). Diet planning in the third world by linear and goal programming. J Opl Res Soc.

[26] Britten P, Marcoe K, Yamini S, Davis C (2006). Development of food intake patterns for the MyPyramid Food Guidance System. J Nutr Educ Behav.

[27] US Department of Agriculture, Center for Nutrition Policy and Promotion. MyPlate. Available online at: http://www.choosemyplate.gov. Accessed April 15, 2014.

[28] Health Canada. Eating Well with Canada’s Food Guide. Available online at: http: www.healthcanada.gc.ca foodguide. (Accessed April 10, 2014).

[29] Ministry of Health, Labour, and Welfare of Japan (2000). The National Health and Nutrition Survey in Japan, 1998.

[30] Ministry of Health, Labour, and Welfare of Japan (2009). The National Health and Nutrition Survey in Japan, 2006.

[31] Livingstone MBE, Black AE (2003). Markers of the validity of reported energy intake. J Nutr.

